# Modeling the effect of operator and passenger characteristics on the fatality risk of motorcycle crashes

**DOI:** 10.5249/jivr.v8i1.650

**Published:** 2016-01

**Authors:** Ali Tavakoli Kashani, Rahim Rabieyan, Mohammad Mehdi Besharati

**Affiliations:** ^a^Faculty of Civil Engineering, Iran University of Science & Technology, Tehran, Iran.

**Keywords:** Motorcycle crashes, Pillion passenger, Injury severity, Logistic regression

## Abstract

**Background:**

In Iran more than 25% of crash fatalities belong to motorcycle operators and passengers in the recent years, from which about 20% are related to passenger fatalities.

**Methods:**

The aim of this study was to investigate the motorcycle operator and passenger characteristics as well as other contributory factors that may affect the fatality risk of motorcyclists involved in traffic crashes. To this end, motorcycle crash data between 2009 and 2012 was extracted from Iran traffic crash database and a logistic regression analysis was performed to obtain odds ratio estimates for each of the study variables.

**Results:**

The fatality risk of motorcyclists has a direct relationship with the number of pillion passengers carried. Results also indicate that the amount of increase in the likelihood of having a fatality in a motorcycles crash is considerably higher when the operator is accompanied by a male passenger of the same age. Furthermore, results showed that if the crash is occurred in the darkness, on curves, in rural areas and on highways, then the crash would be more likely to be fatal. Moreover, the head-on collisions, older operators, unlicensed operators and not using a safety helmet were found to increase the likelihood of a fatality in a motorcycle crash.

**Conclusions:**

Preventative measures such as, imposing stricter rules regarding safety helmet usage and confining the number of pillion passengers to one, might be implemented to reduce the fatality risk in motorcycle crashes. In addition, more appropriate infrastructures for penalizing offending motorcyclists could also reduce the frequency of law violations such as not wearing helmet or riding without motorcycle license, which in turn, would result into a reduction in the fatality risk of motorcycle crashes.

## Introduction

Motorcycles are one of the most popular modes of transportation in Iran. This might be attributed to several factors including affordable price of a motorcycle, easier parking conditions and higher maneuverability of motorcycle, particularly in the dense traffic conditions. On the other hand, the severity of motorcycle crashes is rather high compared to other types of motor vehicles due to the low stability of the motorcycle and lack of protection for the passengers.^1^ Consequently, motorcyclist are involved in a significant proportion of fatal crashes all over the world. According to the Iran Legal Medicine Organization report in 2011, more than 5000 motorcyclists have been died due to traffic crashes, which is more than 25% of all traffic casualties in Iran. Hence, identifying the factors affecting the fatality risk of motorcyclists is an important task which can help to reduce the death rate of this road user group.

So far, the effect of various factors on the injury severity of motorcyclists has been investigated. These factors can be categorized into four groups: human factors, vehicle factors, crash characteristics, roadway and environmental factors. 

The human factors include the operator characteristics such as age, gender, riding license, safety helmet usage and alcohol consumption. The results of several studies confirm that the crash severity is significantly related to increasing operator age.^2-4^ For example, Jou et al. argued that higher fatality rates among motorcyclists in Taiwan correlate with male, older, unlicensed riders.^2^ Safety helmet usage is another human factor which plays an important role in saving the lives of motorcycle operators and passengers.^5-8^ In addition, several studies conducted in Taiwan,^2,9^ United States^3^ and Thailand^10^ have found that the likelihood of a fatality in a motorcycle crash was significantly increased when the operator was an unlicensed male or had consumed alcohol.Moreover, several previous studies have investigated the effect of operator gender on the injury severity of motorcyclists. However, different results were reported by each of the researchers. For instance, in the studies conducted by Quddus et al. in Singapore and Rifaat et al. in Calgary, Canada, the severity of crashes were reported to be higher for female operators.^11,12^ However, Jou et al reported a higher risk for male operators. ^2^

In the term of vehicle characteristics affecting the fatality risk of motorcyclists, riding heavy motorcycles was found to significantly increase the severity of motorcycle crashes.^2,3^

Additionally, the crash characteristics such as crash type (i.e., single or multiple-vehicle crash) and crash mechanism (i.e., head-on collisions or rear-end collisions) are also influential factors associated with severity of crashes. For example, Savolainen and Mannering have studied the factors affecting the motorcyclists' injury severity in single- and multi-vehicle crashes occurred in United States and argued that if the crash is caused due to head-on collisions in multiple-vehicle crashes, the passenger injury severity is approximately 6 times more likely to be fatal.^3^

Finally, roadway and environmental factors such as geometric design, road type, pavement conditions, weather condition, area type (i.e., urban or rural) and illumination were suggested to affect the severity of motorcycle crashes.^1,13,14^ For instance, results of a study conducted in Myanmar have indicated that the crashes occurred in rural areas, suburban regions and also in dark conditions are more likely to be fatal.^14^ The severity of crashes was also reported to substantially reduce in bad weather and wet pavements, particularly in winters.^3,12,15^

Although several previous studies have investigated the effect of the presence of passenger on the crash severity of motorcyclists,^2,3,11^ very few studies have focused on the passenger characteristics such as age, gender and number of passengers that might contribute to the injury severity of these crashes. In the present study, we aim to classify the passengers into homogeneous classes based on their characteristics and investigate the effect of their characteristics as well as some other contributory factors on the likelihood of having a fatality in a motorcycle crash.

## Methods

To investigate the influence of the factors on the severity of motorcycle crashes, the dependent variable is categorized into two levels of fatal (case) and non-fatal (control). Regarding the type of dependent variable in the present study, the Binomial Logistic Regression was employed to model the effect of each variable on the variation of probabilities of crashes being fatal. Therefore the probability of motorcycle crashes to be fatal is obtained through the following equation:

LN(P⁄1-P)=a+β_1_ X_1_+β_2_ X_2_+...+β_k_ X_k_ (1)

Where P is the probability of motorcyclist injury severity to be fatal, 1-P is probability of the injury severity not to be fatal; X_i_, denote the independent variables and β_i_ are the set of regression coefficients that indicate the varying amount of logarithms of probability of a crash being fatal per unit change in each variable. In logistic regression, the odds ratio is used to evaluate the amount of influence of any independent variable on the dependent variable. This statistic measures the chance of occurrence of an event due to a unit change in the independent variable and will be equal to EXP (β_i_) according to the model.

In order to investigate the significance of independent variables in the model, Wald statistic which has the same interpretation asthe t-statistic in linear regressions, was calculated. In fact, these statistics test the null hypothesis that all regression coefficients are zero. Thus, if the null hypothesis is rejected, the independent variable is said to be statistically significant.

In the present study, the Receiver Operating Characteristics (ROC) curves and accuracy measurements is employed to assess the performance and validation of logistic regression models. These concepts are both computed based on classification table. As previously stated, the aim is to classify the dependent variable in two levels (e.g. positive and negative), thus the model will classify the samples in two categories based on the predicted probabilities for any sample and the determined cut-off point. Accordingly, four different cases are summarized in Table 1.

**Table 1:** Classifying the model according to the model prediction.

Observed	Predicted	
	Positive	Negative
**Positive**	TP	FP
**Negative**	FN	TN
**Total**	P	N

In Table 1, TP and TN, denote the number of positive and negative samples respectively which have been accurately predicted by the model. In return, FP and FN indicate the number of positive and negative samples which have been wrongly classified. To assess the model’s performance using the classification table, the following measures need to be calculated first:

Sensitivity=TPR= TP⁄P(2)

1-Specificity=FPR= FP⁄N (3)

Accuracy= ((TP+TN))⁄((P+N))(4)

ROC is a two-dimensional diagram in which the horizontal axis is the FPR and the vertical axis is the TPR. To draw the diagram, the TPR and FPR values of the cut-off points between 0 and 1 are calculated using Equation 2 and Equation 3. A curve is then fitted by connecting the points. The area under the curve which always varies between 0.5 and 1, is considered as an appropriate measure to investigate the model’s performance in classifying the samples. If the area under the ROC curve is greater than or equal to 0.9, the model is considered to have outstanding discrimination. Furthermore, if the area under the curve is between 0.9 and 0.8, the model is considered to have excellent discrimination and finally if the area under the curve is between 0.8 and 0.7, the model is considered to have acceptable discrimination.^16^

The accuracy of the model is obtained through Equation 4. An accuracy value close to or equal to 1 confirms the high power of the model to classify the samples. An important point in calculating the accuracy is its varying value as the cut-off points vary. It should be noted that the TP and TN values vary conversely, as the cut-off points vary. Accordingly, the optimal cut-off point is the one which predicts the maximum values for both TP and TN. It worth mentioning that Two methods have been suggested in the literature to obtain the optimal cut-off point using ROC curve: 1) the optimal cut-off point in ROC curve is the one in which the sum of sensitivity and specificity yields the maximum value 2) the optimal cut-off point in ROC curve is the one which has the minimum distance with the optimal point. The optimal point is the one in which all samples are accurately classified in their own category. In such case the TPR and FPR values are 1 and 0 respectively. The distance of all curve points to the optimal point is obtained through Equation 5. In the present study, both methods were employed and they yield the same results.

d=((1-TPR)^2^+(FPR)^2^)^(1⁄2)^ (5)

Two criteria including the area under ROC curve and classification accuracy can be applied to validate the models. To this end, 70% of the data was randomly chosen for training the model and the remaining 30% was used to test the model. The model obtained from training data, is fitted to the testing data. Hereby, the model can be validated through two methods: the first is to compare the classification error for both testing and training data. In case there is a significant difference, the model would not be strong enough to be employed for prediction.^17^ The second method is to compare the area under ROC curve with the standard deviation related to testing and training data. Similar to the former, if there is a significant difference between the results obtained from the training and testing data, the model would not be appropriate enough to be used for prediction application. ^18^

In this study the model was built based on a stepwise procedure called the Forward Likelihood Ratio in which each variable enters the model based on the significance of the likelihood ratio and exits according to their probability and estimation of partial maximum likelihood. Once the significant variables and the optimal cut-off points were determined using the results of Forward Likelihood Ratio, a new model including the significant variables was fitted to the data using the Enter method.

**Crash Data**

For this study, crash data maintained by the Iran Traffic Police from 2009 to 2012 have been used. To obtain the data relevant to this study, the data pertaining to motorcycle crashes were extracted from the original database. Finally 127995 motorcycle-involved crashes were identified for the analysis. 

The dependent variable in the present study is the injury severity of motorcyclist which is classified into two classes of fatal and non-fatal. Motorcycle fatal crash is defined as at least one motorcyclist killed on the event of road crash. To investigate the effect of pillion passenger characteristics on the severity of crashes, pillion passengers were divided into four groups according to their age and gender. To do this, the passenger age, was first classified into two groups compared with the operator age: in the same age (in the range of ±5 years) and not in the same age (more than 5 years). Each age group was then classified based on gender into two classes as male and female.For motorcycles carrying three people (an operator and two pillion passenger), two typical groups were identifiable in the database that can be characterized as “operator and two male passengers in the same age” and “operator and his wife and their little child”. To distinct these two groups from others, two indexes were considered to classify the passengers: 1) the maximum age interval between the operator and passengers and 2) the gender of the oldest passenger. Accordingly, the passengers were classified into three groups: 1) male in the same age 2) female not in the same age and 3) others. Finally, due to the low number of crashes with four or more people on one motorcycle, we eliminate them from the model. The percentage and number of fatal and non-fatal crashes according to different levels of variables under study are shown in Table 2.

**Table 2:** Percentage and frequency of fatal and non-fatal crashes according to different level of variables.

Variable	Level	Number of Fatal	percent of fatal	Number of non-fatal	percent of non-fatal
**Operator characteristics**					
****	Teenage (12-19)	363	1.58	22595	98.42
****	Young(20-29)	883	1.44	60449	98.56
**Operator age**	Middle-aged(30-39)	412	1.69	23915	98.31
****	Adult(40-50)	255	2.09	11970	97.91
****	Old(51-100)	248	3.47	6905	96.53
****	No	385	1.22	31138	98.78
**Riding license**	Unknown	1735	2.27	74859	97.73
****	Yes	41	0.21	19837	99.79
**Passenger characteristics**					
****	One male in the same age	202	2.79	7037	97.21
****	One female in the same age	29	1.62	1764	98.38
****	One male not in the same age	93	2.31	3937	97.69
****	One female not in the same age	37	1.76	2069	98.24
**Passenger attitude**	Two-passengers (male in the same age)	23	5.31	410	94.69
****	Two-passengers (female not in the same age)	16	2.93	531	97.07
****	Two-passengers (others)	15	4.57	313	95.43
****	No passenger	1746	1.57	109773	98.43
****	At least one passenger did not use	1287	2.35	53449	97.65
**Safety helmet**	All passengers used	112	0.97	11412	99.03
****	Unknown	762	1.23	60973	98.77
**Time of crash**					
****	7:00 AM-7:00 PM	1303	1.47	87158	98.53
**Day time**	7:00 PM- 00:00 AM	659	1.91	33882	98.09
****	00:00 AM-7:00 AM	199	3.99	4794	96.01
**Crash Characteristics**					
****	Intra-city highway	121	5.68	2011	94.32
****	Intra-city main roads	557	5.36	9830	94.64
****	Intra-city local roads	462	4.67	9439	95.33
****	Intercity highway	83	1.52	5378	98.48
**Road type**	Intercity main streets	585	0.78	74520	99.22
****	Intercity local streets	90	0.71	12667	99.29
****	Rural roads	56	3.43	1576	96.57
****	Unknown	207	1.95	10413	98.05
****	Curve	247	4.40	5364	95.60
**Geometry**	Straight	1691	1.51	110238	98.49
****	Unknown	223	2.13	10232	97.87
**Collision characteristics**					
****	Pedestrian/bicycle	29	0.28	10447	99.72
****	Single-vehicle	421	8.18	4724	91.82
**Crash type**	Multiple-vehicle	1693	1.52	109827	98.48
****	Unknown	18	2.18	806	97.82
****	Head-on	539	2.80	18729	97.20
****	Rear-end	323	1.90	16713	98.10
**Crash mechanism**	Sideswipe	615	0.98	61984	99.02
****	Others	684	2.35	28408	97.65

## Results

Table 3 indicates the results of model assessment for training and testing data. The values related to the area under ROC curve and the accuracy of the model constructed by training data indicate the convincing performance of the model in classifying the samples into two groups of “fatal” and “non-fatal”. Furthermore, by comparing the results of model assessment shown in Table 3, it can be seen that a little difference exists between the standard deviation of the two models constructed with the training and testing samples. The accuracy and the area under the ROC curve are also the same for the two models. Therefore, the model is valid enough to be applied for prediction. The model parameters are shown in Table 4. The level of each variable which is not indicated in the table is regarded as the reference level with which the other levels are compared.

**Table 3:** The results of model assessment (area under the ROC curve, standard deviation and model accuracy).

Model	ROC curve			AC1	PER2
	Area	Std. error	Sig.		
**Training data**	0.821	0.005	0.000	0.784	0.216
**Testing data**	0.799	0.009	0.000	0.768	0.232

1 Accuracy (cutoff=.019) 2 Prediction Error Rate=1-AC

**Table 4:** The results of modeling to determine the effects of variables on fatality of motorcycle crashes.

Variable	Level	EXP(β)	95% C.I1	
****	Teenage (12-19)	.86	.737	.993
****	Young(20-29)	.84	.740	.945
**Operator age**	Adults(40-50)	1.2	1.017	1.405
****	Old(51-100)	2	1.692	2.355
**Riding license**	No	4.33	3.124	6.006
****	Unknown	6.38	4.664	8.37
****	One male in the same age	1.79	1.532	2.094
****	One female in the same age	1.04	.711	1.519
****	One male not in the same age	1.43	1.147	1.772
**Passenger attitude**	One female not in the same age	1.11	.795	1.557
****	Two-passengers (male in the same age)	3.47	2.227	5.504
****	Two-passengers (female not in the same age)	2.01	1.201	3.375
****	Two-passengers (others)	3.01	1.765	5.144
**Safety helmet**	At least one passenger did not use	2.56	2.096	3.119
****	Unknown	1.46	1.189	1.784
**Day time**	7:00 PM- 00:00 AM	1.23	1.113	1.351
****	00:00 AM-7:00 AM	2.35	2.006	2.760
****	Intra-city highway	4.79	3.887	5.908
****	Intra-city mail roads	4.82	4.253	5.453
****	Intra-city local roads	3.96	3.446	4.513
**Road type**	Urban Highways	1.78	1.406	2.259
****	Urban local streets	.855	.683	1.069
****	Rural road	3.04	2.283	4.043
****	Unknown	2.15	1.814	2.539
**Geometry**	Curve	1.35	1.164	1.557
****	Unknown	.96	.820	1.116
****	Pedestrian or bicycle	.25	.174	.336
**Crash type**	Single-vehicle	2.98	2.591	3.437
****	Unknown	1.26	.770	2.052
****	Head-on	2.28	2.017	2.567
**crash mechanism**	Rear-End	1.50	1.308	1.728
****	Unknown	1.46	1.280	1.674
**Chi-square(DF2=54)**		**3136.802**		

1confidence interval 2 Degrees of freedom

Note that the odds ratio for the middle-aged operator is selected equal to 1. As shown in Table 4, while the fatality risk decreases for the younger operators, it increases for older ones. In other words, there is a direct relationship between the operator age and the fatality in a way that the increase of age leads to an increase in the fatality risk of motorcyclists. One reason could be the physical weakness and much vulnerability of older operators.^3^ The results also indicate that the fatality risk of crashes for teenage and young operators is almost the same. In return, the fatality risk for older operators shows a considerable differences with other age groups which is another indicator of the underlying influence of physical weakness on crash severity. This is in line with the findings of previous studies.^2-4,13^

Previous studies also indicate that riding license is another influencing factor that has a key role on the fatality risk of motorcyclists.^1,19^ Based on the Table 4, the fatality risk would be four times greater if the operator is unlicensed. Having less experience and riding skills which in turn leads to an increase in the operator’s reactiontime might be the reason of such an increased risk.^20^ Another reason could be the associations among human factors. For example, the unlicensed operators might be more likely to ride with higher speeds and less likely to wear safety helmets.^21,22^

Based on Table 4, it can be inferred that if at least one of the motorcyclists (either operator or passenger) does not wear a helmet, the crash would be 2.5 times more likely to be fatal. Since most of the reported motorcycle fatalities are due to head trauma, wearing a safety helmet can help to reduce the death rate of motorcycle crashes. This is consistent with findings of previous studies.^2,3,6,23,24^ For example, in their study Jou et.al have argued that not wearing a helmet increases the likelihood of fatal crashes to 280%.^2^

In the next step, the effect of passenger-related factors on the fatality risk of motorcycle crashes were investigated. The odds ratio equal to 1 was assumed for motorcycles with no passenger and the relative risk of other groups was determined accordingly. According to Table 4, it is evident that the fatality risk increases by increasing the number of pillion passengers. The lowest risk is found for the case of “no passenger” and the highest for motorcycles with 2 pillion passengers. This might be because of the higher number of people exposed to danger in this situation. ^11^ Similar studies obtained different results considering the effect of pillion passengers on the probability of motorcycle crashes being fatal. For example, while the results of the study conducted by Jou et al. indicate the reversed relationship between the number of passengers and fatality risk of motorcyclists, other studies indicates a direct relationship in similar case. ^25,26^

Table 4 also reveals the effect of passenger gender on the fatality of motorcyclists. As can be seen, for both groups of one-passenger and two-passenger, the lowest risk belongs to females regardless of their age-group, in a way that there is no significant difference between the fatality risk of motorcycle crashes with no passenger and those with one female passenger. Considering the fact that the female passengers in Iran commonly have an intimate relationship with the motorcycle operators, therefore, much responsibility of the operators toward such passengers might encourage them to move with lower speeds and higher caution. Consequently, the odds of fatal crash would substantially decrease in these cases. Another important result obtained from Table 4 relates to the effect of passenger age on the fatality risk of motorcycle crashes. In the case of “male” passenger with the same age as operator (i.e., within the range of ±5 years), the fatality risk increases but the female passenger has no considerable influence on the corresponding fatality risk of the crash. 

## Discution

According to Table 4, the crash characteristics such as crash type and crash mechanism also affect the fatality risk of crashes. The odds of crash being fatal decreases about 75% if motorcycles collide with pedestrians or bicycles. This means that pedestrian and bicyclists are more vulnerable than motorcyclists which was already predictable. This is consistent with the findings of similar studies. ^11,24^ Unlike the collision with a pedestrian, the one-vehicle motorcycle crashes are three times more likely to be fatal compared with the multiple-vehicle crashes. On the contrary, several other studies indicate the higher severity of crashes resulting from multiple-vehicle crashes compared with single-vehicle crashes.^2,13^ This might be due to the fact that a considerable extent of single-vehicle motorcycle crashes are not reported to the Traffic Police because the insurance system in Iran does not cover the motorcycle crashes.

Day time is another factor that might affect the severity of crashes. Classifying the crash time in the present study is based on the illumination. The rate of increase in crash severity is about 23% at early night (7:00 PM to 12:00 AM) and 135% at late night (12:00 AM to 7:00 AM). Similarly, a number of previous studies have reported that the likelihood of having a fatality in a crash increases at darkness. ^2-4,12,24^

In the current study, road classification was based on two criteria: 1) the road function, 2) area type in the vicinity of the road. By considering the odds ratio for the intercity main streets equal to 1, the relative odds for other roads are demonstrated in Table 4. As can be seen in this table, the fatality risk of crashes on rural roads is considerably higher compared with the urban areas. The lower risk of fatal crashes in urban areas could be explained by denser traffic flow in these areas as well as absence of traffic light, less police control, darkness and much more frequent presence of trucks in the rural roads. This confirms the results of previous studies. ^2,4,13^

The road type might be regarded as another factor that contributes to the increase in the fatality risk of motorcycle crashes. As one can see in Table 4, the motorcycle crashes are more likely to be fatal for the crashes occurred on highways. This might be due to higher relative speeds in these road types.

The roadway geometric design is another factor which has a significant influence on the fatality risk. Based on the results, the odds of fatality in motorcycle crashes occurred on curves is 35% higher than those occurred on straight segments and this is in line with the findings of previous studies. ^3,4^

So far, very few studies have been conducted to investigate the interaction effect of passenger age and gender on the severity of motorcycle crashes. Therefore, we compare the results of this study with previous studies on passenger car crashes. Previous studies on car crashes indicate that if the passengers are male and in the same age as driver, then the young male drivers will drive faster than the general traffic and allow shorter headways.^25,27-29^ As an example, Lam et.al have showed that the severity of crashes for drivers younger than 25 years, accompanied by a passenger of the same age, is 2.39 times greater and if the driver is accompanied by two or more passengers of the same age, the corresponding risk would be 15.55 times greater. The situation seems to be similar for motorcycle operators. Since in the present study, most of the recorded crashes are related to the teenage and young operators, riding with higher speeds and allowing shorter headways can be the cause of the increased fatality risk of the “one male passenger in the same age” group (see Table 4).

## Conclusion

This paper investigates the effect of several contributory factors on the likelihood of having a fatality in the motorcycle crashes. According to the results, crashes occurred during darkness, on curve, in rural areas and on highways are more likely to be fatal. The fatality risk is also higher for head-on collisions and older motorcycle operators. Having no riding license and not wearing helmets are also two influential factors which increase the fatality risk of motorcycle crashes. 

In addition, the interaction effect between age and gender of the operator and passengers was found to significantly influence the fatality risk of motorcyclists. For instance, if the operator is accompanied by a male passenger of the same age, then the crash would be more likely to be fatal.Moreover, the presence of pillion passenger increases the probability of a crash being fatal. Additionally, the number of pillion passengers was found to have a direct relationship with the likelihood of having a fatality in motorcycle crashes. In other words, results indicate that a motorcycle crash is less likely to be fatal if there are no passengers on the vehicle. Thus, based on the finding of this study, legislations restricting the number of passengers to no more than one would help decrease the fatalities related to motorcycle crashes.

Furthermore, motorcycle operators and passengers should be informed about the risk of not using a proper helmet and carrying more than one pillion passenger on a motorcycle. This might encourage them not to ride a motorcycle with more than one passenger and use a helmet for each passengers.

Finally, the authors believe that more appropriate infrastructures for penalizing offending motorcyclists could reduce the frequency of law violations such as not wearing helmet or riding without motorcycle license, which in turn, would result into a reduction in the fatality risk of motorcycle crashes.

**Acknowledgment**

The authors would like to extend their very special thanks and appreciation to Mr. Sabbaq and Mr. Mishani for their relentless genuine collaboration in providing the data.
